# CADBURE: A generic tool to evaluate the performance of spliced aligners on RNA-Seq data

**DOI:** 10.1038/srep13443

**Published:** 2015-08-25

**Authors:** Praveen Kumar Raj Kumar, Thanh V. Hoang, Michael L. Robinson, Panagiotis A. Tsonis, Chun Liang

**Affiliations:** 1 Department of Biology, Miami University, Oxford, Ohio 45056, USA; 2 Department of Biology and Center for Tissue Regeneration and Engineering, The University of Dayton, Dayton, OH 45469; 3 Department of Computer Sciences and Software Engineering, Miami University, Oxford, Ohio 45056, USA

## Abstract

The fundamental task in RNA-Seq-based transcriptome analysis is alignment of millions of short reads to the reference genome or transcriptome. Choosing the right tool for the dataset in hand from many existent RNA-Seq alignment packages remains a critical challenge for downstream analysis. To facilitate this choice, we designed a novel tool for comparing alignment results of user data based on the relative reliability of uniquely aligned reads (CADBURE). CADBURE can easily evaluate different aligners, or different parameter sets using the same aligner, and selects the best alignment result for any RNA-Seq dataset. Strengths of CADBURE include the ability to compare alignment results without the need for synthetic data such as simulated genomes, alignment regeneration and randomly subsampled datasets. The benefit of a CADBURE selected alignment result was supported by differentially expressed gene (DEG) analysis. We demonstrated that the use of CADBURE to select the best alignment from a number of different alignment results could change the number of DEGs by as much as 10%. In particular, the CADBURE selected alignment result favors fewer false positives in the DEG analysis. We also verified differential expression of eighteen genes with RT-qPCR validation experiments. CADBURE is an open source tool (http://cadbure.sourceforge.net/).

RNA-Seq analysis describes the collection of methods to characterize transcriptomes using high-throughput sequencing of abundant cDNA fragments^1^,^2^. The fundamental task in RNA-Seq-based transcriptome analysis is spliced alignment (called aligning or mapping) of short sequencing reads to the reference genome or transcriptome^1^,^2^,^3^. The complexities inherent in RNA-seq data require specialized tools (i.e., aligner) to map them to genome. RNA-seq data possesses many complexities like alternate splice isoforms that can contain many different combinations of exons transcribed from a gene^2^,^4^. As with many high-throughput sequencing technologies, the sequence fragments are short (35 nt to 100 nt), presenting considerable challenges to an aligner which must determine the unique mapping location among the many possible locations in the reference genome^5^,^6^. Furthermore, the reference genome is most often different than that of the actual biological source of RNAs being sequenced. Organism-specific sequence properties like repetitive sequences, gene structures, polymorphisms, and RNA polymerase fidelity compound the difficulty in achieving proper alignment of a given sequence read. In these circumstances, the aligner’s solution is often a compromise between the sensitivity and specificity provided an initial level of heuristic match^2^,^7^,^8^,^9^,^10^,^11^. This tradeoff in specificity and sensitivity makes an aligner work better on some datasets than others.

In RNA-Seq projects, one major challenge is to choose a right mapping tool among many existing tools. Current tools available for mapping sequencing reads include GSNAP^9^, TopHat2^8^, CLC Genomics Workbench (CLC Bio-Qiagen, Aarhus, Denmark), MapSplice^11^, PALMapper^12^, STAR^13^ to name a few (for an exhaustive list see^14^). Although many previous investigations compared different aligners^3^,^8^,^9^,^11^,^13^,^15^, to our knowledge, no single aligner demonstrates clear superiority in the critical performance metrics of speed, accuracy and correct mapping placement. Moreover, the performance of an aligner depends on specific properties of the dataset being analyzed. These properties include repeat content, intron-exon architecture, sequence polymorphisms such as insertions, deletions and mismatches, sequence coverage, and sequencing error rate^2^,^3^. As a result, aligner choice, or even the parameter set for a specific aligner needs to be evaluated in the context of the dataset of interest. This is not a trivial task since it may be impossible to *a priori* determine which aligner or parameter set best matches the real situation when evaluating an aligner or a parameter set on real data.

A recently published alignment evaluation tool, ARDEN^16^ proposes a solution to comparing aligners on a given dataset. However, ARDEN requires time-consuming genome simulation and random data subsampling in pre-processing steps. ARDEN also requires alignment regeneration against a simulated genome using the same parameter set used for generating the original alignments^16^. This step is impractical because genome-specific options of the original reference genome (e.g., gene-annotation guided mapping and SNP-tolerant mapping) cannot be used against a simulated genome. To create an easy-to-use mapping comparison utility that avoids time-consuming and complex pre-processing steps, we developed a tool for comparing alignment results of user data based on the relative reliability of uniquely aligned reads (CADBURE). CADBURE (pronounced “Cadbury”) compares alignment results obtained from two different aligners or two different parameter sets of a single aligner. The alignment comparison in CADBURE is independent of the parameter set used, and does not require alignment regeneration, random dataset subsampling and genome simulation. CADBURE bases its alignment comparison on identifying the relative reliability of uniquely mapped reads between two different alignment results.

The use of CADBURE was demonstrated by comparing alignment results of 6 RNA-Seq datasets^17^ from two different mouse lens cell-types. CADBURE found the optimal alignment result among the 3 widely used splice alignment programs: two open source aligners GSNAP and TopHat2, and a commercial software package CLC Genomics Workbench. To demonstrate the reliability of CADBURE in selecting the optimal alignment result, we performed downstream analysis to compare the number of differentially expressed genes (DEGs) identified using the CADBURE selected alignment with alignments not selected by CADBURE. To decide on the method for DEG analysis, we used the CADBURE selected best alignment result to evaluate the performance of five popular tools for DEG analysis: (1) DESeq^18^, (2) DESeq2^19^, (3) Baggereley test, CLC version 6.5.1, (4) Empirical analysis of differential gene expression (EDGE), CLC version 7.0 and (5) Cuffdiff2^20^. To our knowledge, this is the first comparative evaluation involving DESeq2 and the popular commercial CLC software. The RT-qPCR verification of 18 DEGs in the datasets revealed that DESeq results closely correlated with RT-qPCR data. Hence, DESeq was selected for DEG analysis to demonstrate the reliability of the CADBURE selected alignment. Comparing the number of DEGs identified using the CADBURE selected alignment result versus other non-selected alignment results, we discovered differences of approximately 10% in the number of DEGs, which may be important in the biological context. Thus, CADBURE provides biologists with a novel, easy-to-use tool for comparing alignment results of a given RNA-Seq dataset in order to select the best or optimal alignment.

## Results

### Design of CADBURE

**Figure 1:**
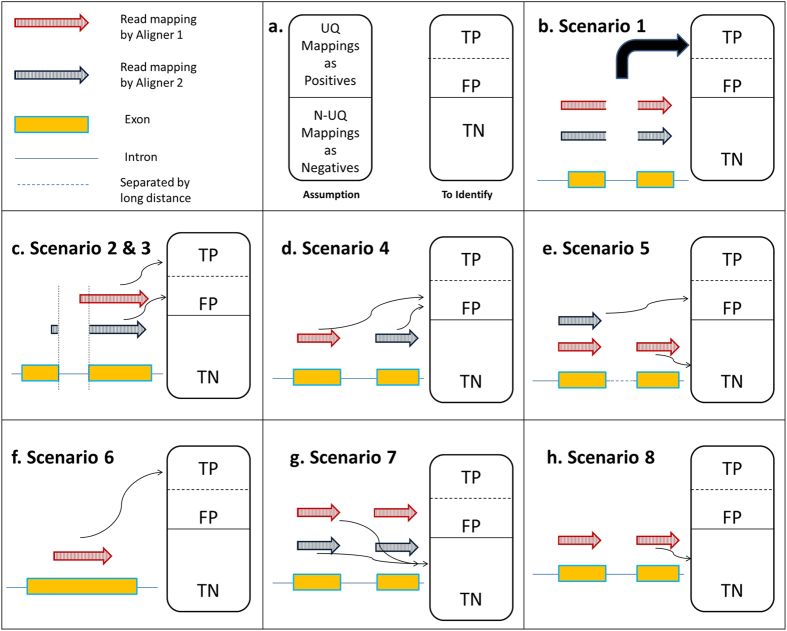
The core algorithm of CADBURE. (**a**) The assumption of CADBURE core algorithm and metrics to identify. The collection of UQ (unique) mappings are assumed as a set of positives and the collection of N-UQ (non-unique) mappings are assumed as a set of negatives. (**b**) Scenario 1. UQ mapping by both aligner-1 (red arrow) and aligner-2 (blue arrow) to the same genome location with the same alignment. (**c**) Scenario 2 & 3. UQ mapping by both aligner-1 and aligner-2 to overlapping genome regions with different alignments. Scenario 2: No spliced alignment by aligner-1 is called as TP (true-positive). Scenario 3: spliced-alignment by aligner-2 with a nucleotide overhang is called as FP (false-positive). (**d**) Scenario 4. UQ mapping by both aligner-1 and aligner-2 to different genomic locations, and both are called FP. (**e**) Scenario 5. UQ mapping by aligner-2 is called FP because the same read is reported as N-UQ mapping by aligner-1. The read mapping in the aligner-1 is called TN (true-negative). (**f**) Scenario 6. UQ mapping by aligner-1 is called TP because aligner-2 cannot map this read as either UQ or N-UQ mapping. (**g**) Scenario 7. N-UQ mapping by both aligner-1 and aligner-2 where they agree for a read in that they map it non-uniquely. This case is called TN for both aligners. (**h**) Scenario 8. N-UQ mapping by aligner-1 is called TN because aligner-2 cannot map this read as either UQ or N-UQ read. UQ: Unique, N-UQ: Non-Unique, TP: true-positive, FP: false-positive, TN: true-negative.

The purpose of CADBURE is to evaluate spliced aligner performance for user’s RNA-Seq data by comparing a pair of alignment results obtained either from two different aligners with the similar parameter set or from two different parameter sets with the same aligner. In alignment comparison, CADBURE determines the relative reliability of unambiguously (also called uniquely) aligned reads. These uniquely mapped reads form the primary input for downstream analysis in RNA-Seq projects^1^,^2^,^21^,^22^. In an alignment result, CADBURE defines the collection of uniquely mapped reads as a positive set and the collection of ambiguously (also called non-uniquely) mapped reads as a negative set in order to provide a discrete measure of specificity and accuracy (Fig. 1a). Then, CADBURE determines true-positives and false-positives among the uniquely mapped reads (i.e. our positives) by evaluating the possible scenarios that can occur with respect to the relative mapping reliability. All the negatives (non-uniquely mapped reads) are assumed as true-negatives. It should be noted that we are not trying to find false negatives in the negatives set (non-unique mapping) because our focus is to find false positives only in the positive set (unique mapping), which is mostly used for downstream analysis in RNA-Seq studies^1^,^2^,^21^,^22^.

As CADBURE compares the alignment of a given read by two different aligners, one of eight scenarios may occur, described below (Fig. 1).

### Scenario 1 (mappings in the positive set)

Whenever two different aligners uniquely map a particular read to the exact same location, CADBURE considers the result a true-positive mapping for an aligner (Fig. 1b).

### Scenario 2 and 3 (mappings in the positive set)

When two different aligners uniquely map a particular read to overlapping genomic locations, CADBURE distinguishes two scenarios.

#### Scenario 2

In this case, CADBURE considers the un-spliced alignment or splice alignment with overhang more than two nucleotides a true-positive mapping because all or most nucleotides are continuously mapped by both aligners on the same genome location. Therefore, this is a highly probable correct alignment for this read (see red arrow, Fig. 1c).

#### Scenario 3

If the overhang of the spliced alignment is just one or two nucleotides, i.e., only one or two nucleotides are aligned across an intron, CADBURE considers the spliced alignment as a false-positive alignment, given the high probability that the alignment is misplaced (see blue arrow, Fig. 1c).

### Scenario 4 (mappings in the positive set)

If both aligners uniquely map a particular read to different genomic locations, CADBURE assigns the mapping as false-positive because both aligners missed the possible read mapping reported by the other aligner and incorrectly reported the alignment as unique (Fig. 1d).

### Scenario 5 (mappings in the positive and negative set)

If one aligner maps a particular read uniquely and the other aligner maps the same read non-uniquely, CADBURE considers the uniquely aligned read as a false-positive alignment and the non-uniquely mapped read as a true-negative, non-unique mapping (Fig. 1e).

### Scenario 6 (mappings in the positive set)

If only one of the two aligners uniquely maps a particular sequence read, and if the other aligner fails to map this read at all (either as unique or non-unique), CADBURE considers the unique mapping a true-positive alignment (Fig. 1f).

### Scenario 7 (mappings in the negative set)

If both aligners map a particular read non-uniquely, CADBURE calls the alignment a true-negative, non-unique alignment (Fig. 1g).

### Scenario 8 (mappings in the negative set)

If one aligner maps a particular read non-uniquely and the other aligner fails to map the read as either unique or non-unique, CADBURE considers the alignment true-negative (Fig. 1h).

Finally, CADBURE calculates the specificity and accuracy for each alignment result using the true-positives, false-positives and true-negatives discussed above (see **Methods**). Bootstrapping statistics^23^ (see **Methods**) ascertains the significance of differences between both the accuracy and specificity of alignment results. Higher accuracy and specificity means fewer false-positives. Non-significant difference in the accuracy and/or specificity measures of alignment results implies neither aligner holds a significant advantage over the other.

### Demonstration of the use of CADBURE

To demonstrate the use of CADBURE in aiding the selection of optimal mapping results for a given dataset, CADBURE was applied to our RNA-Seq project^17^ consisting of 6 sequencing samples. The sequenced datasets ranged from 2.4 to 3.3 × 10^7^ single-end reads of length 51 bases (Supplemental Table S1). These samples consisted of three biological replicates from two different cell types (lens epithelial cells and lens fiber cells), from newborn inbred *FVB/N* strain mouse lenses.

#### Mapping to the reference genome

After filtering out low quality reads and trimming off sequencing adapters, mapping was performed against a *Mus musculus* reference genome assembly. A SNP database and gene annotation (ENSEMBL^24^release 72) were utilized to perform a SNP-tolerant and gene-annotation-guided mapping. Altogether, CADBURE evaluated 12 different parameter sets for the different spliced aligners to fully evaluate the aligner’s performance in spliced alignment. These included the variation of 3 different mismatch levels (0, 1 and 2 mismatches) allowed for 3 different aligners: (i) GSNAP, (ii) TopHat2, and (iii) CLC Genomics Workbench. In addition, GSNAP was evaluated with SNP-tolerant option to account for differences between the reference genome and experimental genome, making it the 4th package for evaluation. Consequently, this results in 12 different parameter sets (i.e., 3 mismatch levels for each of the 4 different packages - see **Methods**). CADBURE only evaluated up to 2 mismatches because 3 or more mismatches would represent greater than 5% of the total read length (mean read length after trimming is 47; Supplemental Table S1) and exceed the observed error rate of next generation sequencing^25^. As expected, changes in the allowed mismatch level dramatically affected the alignment yield, ranging from 53.07% to 99.61% (Supplemental Fig. S1 and Supplemental Table S2). As seen in the figure, GSNAP using the maximum 2 mismatches, with and without the SNP-tolerant option, yielded the most consistent mapping for all data samples ranging from 95.47% to 99.61%, followed by TopHat2 ranging from 79.90% to 95.72%. Meanwhile, in comparison to GSNAP and TopHat2 with just 1 allowed mismatch, CLC, surprisingly, demonstrated the lowest yield even with 2 allowed mismatches. The ability of GSNAP to map reads partially by soft clipping ends and aligning reads past annotated exons may be the major reason for many additional mappings with this software.

#### Evaluation using CADBURE

**Figure 2:**
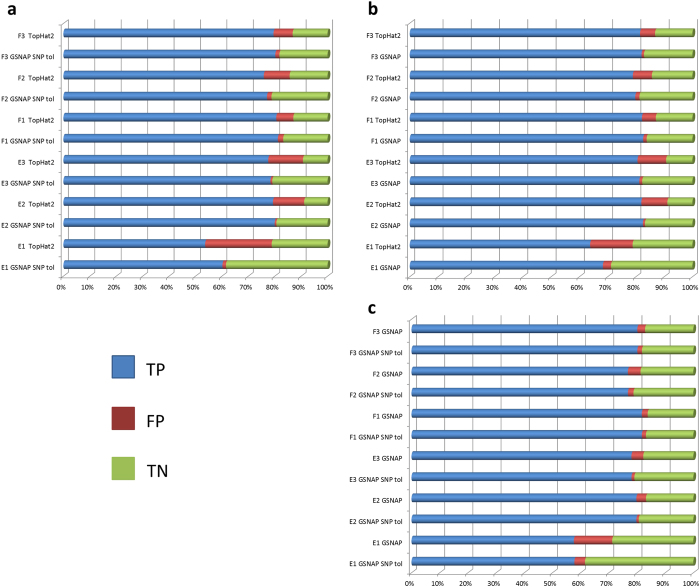
Pairwise comparison of alignment results using CADBURE. (**a**) GSNAP with SNP tolerance versus TopHat2. (**b**) GSNAP without SNP tolerance versus TopHat2. (**c**) GSNAP without SNP tolerance versus GSNAP with SNP tolerance. (**a**–**c**) show the comparison of the percentage of TP, FP and TN for each dataset (E1, E2, E3, F1, F2, F3), where E stands for mouse lens epithelial cells, F for mouse lens fiber cells, and the number indicates the biological replicate number. An asterisk at the end of each sample comparison indicates the difference of both specificity and accuracy between the aligners is significant at 5% level with 95% confidence. GSNAP SNP tol: GSNAP with SNP tolerance.

CADBURE evaluated the top 3 alignment results based on the alignment yield, an important factor in evaluating the optimal alignment result: (1) GSNAP with SNP tolerance and 2 mismatches, (2) GSNAP without SNP tolerance, with 2 mismatches and (3) TopHat2 with 2 mismatches (Supplemental Fig. S1). In the initial comparisons between GSNAP and TopHat2, both GSNAP with and without SNP tolerance produced significantly more reliable mappings among the 8 scenarios (see **Design of CADBURE**) than those from TopHat2 for each of the 6 datasets (Fig. 2a,b; Supplemental Table S3–S6). Next, in the comparison of GSNAP with and without SNP tolerance, the former produced significantly more reliable mappings among the 8 mapping scenarios for all 6 data samples (Fig. 2c; Supplemental Table S7, S8). Thus GSNAP with SNP tolerance and two mismatches consistently produced more reliable mappings for each of the 6 datasets.

#### Validation of CADBURE

**Figure 3:**
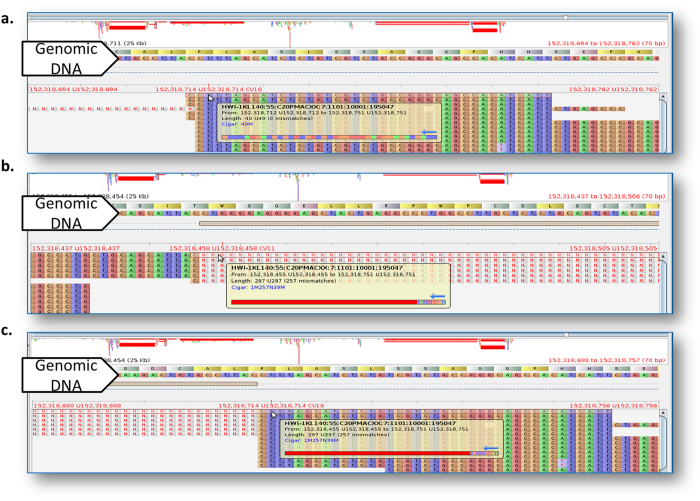
An example of Scenario 2 & 3. Read mapping (highlighted with red line) is identified as Scenario 2 and Scenario 3 by CADBURE and visualized in Tablet^26^. Tablet shows reads mapped against the mouse reference genome. The same read (name shown in popup) is mapped differently to the genome by GSNAP and TopHat2 aligners. (**a**) GSNAP mapped the same 40 base read perfectly with no mismatches to Chromosome 2 from 152,318,712 to 152,318,751. (**b**) TopHat2 mapped the same read with a spliced alignment to Chromosome 2 with 1 base at 152,318,455, leaving a long gap for an intron (257 bp; from 152,318,456 to 152,318,712; displayed with ‘N’) and then (**c**) TopHat2 mapped 39 bases continuously from 152,318,713 to 152,318,751.

CADBURE was validated through verification of the automatic identification of true-positive, false-positive and true-negative by visualizing the read mapping scenarios for alignment result comparisons used above. Figure 3, Supplemental Figs S2 and S3 display the snapshot of read mapping visualized by Tablet^26^ for 3 contradicting read mapping scenarios among compared aligners. The read mapping in Fig. 3 provides an example of scenario 2 and 3 of read mapping (Fig. 1d), where both GSNAP and TopHat2 uniquely mapped the highlighted read to an overlapping genomic location, but with different spliced alignment. GSNAP mapped this 40 base read perfectly with no mismatches to Chromosome 2 from 152,318,712 to 152,318,751 (true-positive), whereas TopHat2 mapped the same read as a spliced alignment to Chromosome 2 with 1 base at 152,318,455, leaving a long gap for an intron (257 bp; from 152,318,456 to 152,318,712) with 39 bases mapped from 152,318,713 to 152,318,751 (false-positive). The read mapping in Supplemental Fig. S2 demonstrates an example of scenario 4 (Fig. 1e), where both GSNAP and TopHat2 aligners uniquely mapped the read to different genomic locations. GSNAP mapped the highlighted 40 base read perfectly with no mismatches to Chromosome 11 from 109,011,648 to 109,011,687 (false-positive; scenario 4), whereas TopHat2 mapped the same read with allowed two mismatches to Chromosome 7 from 110,059,825 to 110,059,864 (false-positive; scenario 4). Here, both GSNAP and TopHat2 missed the possible read mapping found by the counterpart and hence wrongly reported a unique mapping. The read mapping in Supplemental Fig. S3 reveals an example of scenario 5 (Fig. 1f), where TopHat2 mapped the highlighted 51 base read with no mismatches to the mitochondrial chromosome from 7,465 to 7,515 uniquely (false-positive), whereas GSNAP, in addition to mapping to the mitochondrial genome, mapped the same read without mismatches to Chromosome 1 from 24,615,063 to 24,615,663 (non-unique mapping; true-negative).

#### The reliability and benefit of the optimal alignment result selected by CADBURE

To demonstrate the reliability of the optimal alignment result selected by CADBURE, which with our datasets is GSNAP with SNP tolerance option and maximum two mismatches, we performed downstream analysis to determine differentially expressed genes (DEGs) on the CADBURE result versus other non-CADBURE results. To decide on the method for DEG analysis, we first used the optimal alignment result selected by CADBURE to evaluate the ability of five popular methods for DEG analysis: (1) DESeq, (2) DESeq2, (3) Baggereley test from CLC version 6.5.1, (4) Empirical analysis of differential gene expression (EDGE) from CLC version 7.0, and (5) Cuffdiff2. We evaluated these methods through different metrics such as: (1) by comparing and contrasting the number of DEGs identified by each method, (2) performance assessment using receiver operating characteristic (ROC) curve, (3) verification with real time quantitative PCR (RT–qPCR). Through the evaluation of different DEG analysis methods (see Supplemental Results and Discussion, Supplemental Figs S4-S6 and Supplemental Table S9), we find DESeq performs the best in DEG detection. Hence, we used DESeq to compare DEG analysis results obtained from the optimal alignment result selected by CADBURE (i.e., GSNAP with SNP tolerance and maximum 2 mismatches allowed) versus two non-CADBURE results: (1) GSNAP without SNP tolerance, with 2 mismatches, and (2) TopHat2 with 2 mismatches.

**Figure 4:**
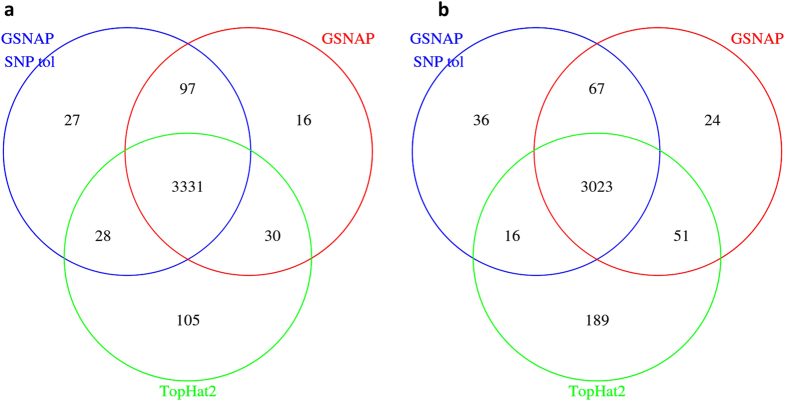
Venn diagrams of Differentially Expressed Genes (DEGs) identified using DESeq from three different alignment results:GSNAP with SNP tolerance and 2 mismatches (CADBURE selected alignment), GSNAP without SNP tolerance, with 2 mismatches and TopHat2 with 2 mismatches. (**a**) DEGs upregulated in Epithelial cells. (**b**) DEGs upregulated in Fiber cells. Significant DEGs are identified with p-adj value <= 0.05.

As shown in Fig. 4, these three alignment results agree in DEG calling for most genes and the fold change for 18 genes with qRT-PCR verification (see Supplemental Results and Discussion and Supplemental Methods) did not differ among them (Supplemental Fig. S7). Although over 88% of both up-regulated (Fig. 4a) and down-regulated genes (Fig. 4b) identified by DESeq as significant DEGs (padj <= 0.05) agree among the three alignment results, there is still a substantial difference among DEG numbers (Fig. 4). Close examinations reveal that the TopHat2 result, which was found to have a higher false positive reads (Figs 1 and 2; section ***Evaluation using CADBURE***), produced more DEGs (Fig. 4) in comparison with the CADBURE result. In particular, we found one DEG, namely Rpl12 (*Mus musculus* ribosomal protein L12; chr2:32,961,712-32,964,045), displays a drastic difference between the results obtained by TopHat2 and GSNAP with SNP tolerance. Namely, the CADBURE selected result reports RPl12 as up-regulated in Epithelial cells whereas TopHat2 reports the same gene as upregulated in Fiber cells (Supplemental Table S10). Supplemental Fig. S8 shows the read alignment snapshot as seen in IGV (Integrative genomics viewer^27^) for the Epithelial replicate 3 (E3) unique reads mapped to gene RPl12 as reported by the CADBURE selected result and TopHat2. Only 650 reads out of 6265 E3 reads reported as uniquely mapped to Rpl12 in TopHat2 are reported as uniquely mapped in the CADBURE selected result (GSNAP with SNP tolerance). Supplemental Table S11 lists all the 6265 E3 reads that are reported in TopHat2 as uniquely aligned to RPl12. Supplemental Table S12 lists the same 6265 E3 reads from the CADBURE selected alignment result, where you can see only 650 of the same reads have been reported as unique alignments. Hence, the difference of RPl12 differential expression reporting in TopHat2 can be mainly attributed to the reads identified as false positives in the TopHat2 mappings (Figs 1 and 2; see the section ***Evaluation using CADBURE***).

## Discussion

The increasing popularity of RNA-Seq-based approaches in transcriptome analysis necessitates a way to evaluate and compare different methods of data analysis. In this work, we introduce CADBURE, a tool to compare and evaluate the performance of alignment programs on any given RNA-Seq dataset. We also demonstrate the use of CADBURE in selecting an optimal alignment result and the reliability of such selected alignment result for downstream DEG analysis. In addition, we provide an evaluation of five different tools used for DEG analysis (Supplemental Results and Discussion). CADBURE satisfies the need for an easy-to-use tool for the comparison of two existing alignment results. Unlike the existing tool - ARDEN^16^, CADBURE comparisons can be independent of the parameters used, and it does not require time-consuming alignment regeneration against a simulated genome or randomly subsampling of the dataset. One of the potential limitations for regenerating alignments against a simulated genome is the preclusion of the use of genome-specific options like gene-annotation or SNP-data guided mapping. CADBURE puts no limitation on the use of these options, eliminating the need for some dispensable preprocessing steps. The only preprocessing step required in CADBURE is the conversion of human-readable SAM (Sequence alignment format) format to machine readable BAM (Binary alignment format) format, which is the input for CADBURE. This SAM to BAM conversion can be easily accomplished using the SAMtools^28^, a package for processing SAM files.

CADBURE is not designed to evaluate the accuracy of read mapping; instead it analyzes the relative reliability and consistency of a read mapping between two alignment programs or parameter sets of the same aligner. The underlying logic of CADBURE is poised to find an aligner that shows optimal performance in terms of the 8 scenarios described in Fig. 1. It should be noted that we are not trying to find false negatives in the negative set (non-unique mapping) because our focus is to find false positives only in the positive set (unique mapping), which is primarily used for downstream analysis in RNA-Seq studies^1^,^21^,^22^. Although CADBURE is designed for pairwise comparison of alignment results, it can be easily used for comparing three alignment results. First, users can compare any two alignment results, and then the CADBURE selected result can be compared with the third alignment result to decide on the optimal one among the three alignments. Our future work will be to increase the comparisons to more than two alignment results automatically.

The demonstration of CADBURE in real data comparison showed the ease of comparing two different alignment results. In our mouse RNA-Seq data, the GSNAP with SNP tolerance option produced more reliable mapping in comparison with other aligners on all of the 6 data samples. However, this result may not hold for other datasets, supporting the strong argument that an aligner must be picked after evaluating different aligners or parameter sets on a particular user’s dataset. In our case, we see the main advantage of GSNAP is its ability to allow mismatches against the reference genome if the read matches the alternate strand of genome and also to map reads partially by soft clipping ends and by aligning reads past annotated exons.

We also demonstrate the reliability of CADBURE-selected alignment results versus other alignment results by comparing the relevant DEGs. Even though we only see about 10% difference among the number of DEGs (Fig. 4) detected among CADBURE result and other alignment results, the CADBURE result favors fewer false positives in DEG calling. As we have shown in Supplemental Figure S8 and Table S11 and S12, the non-unique alignment found for gene *Rpl12* in the CADBURE selected alignment was incorrectly reported as unique in the non-CADBURE selected alignment, which affected DEG profiles adversely. Moreover, we evaluated five popular RNA-Seq-based DEG analysis methods for their ability to discriminate DEGs. Through three different metrics, we found DESeq performed better with better balance between sensitivity and specificity (Supplemental Results and Discussion, Supplemental Figs S4-S6).

The popularity of RNA-Seq-based transcriptome profiling has driven the need for the many different analysis tools from spliced alignment to statistical methods for DEG identification^1^,^2^,^3^. The software comparison illustrated here, along with an introduction of the CADBURE method for alignment evaluation of user data, demonstrates the need for choosing appropriate tools specific to properties of the data. Although the optimal alignment selected by CADBURE results in a lower number of DEGs, CADBURE performs better in reducing false positive mappings. In the comparison of the statistical methods for discriminating DEGs, DESeq performs better in comparison to other tools used in this study.

## Methods

### Accuracy and Specificity by CADBURE

The deduced true positive (TP), false positive (FP) and true negative (TN) map alignments were used to compute the following measures for each alignment result under comparison.
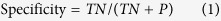

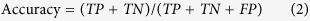


False negative map alignments were not considered in computing accuracy for the reasons presented in the **Results**. Here Specificity (Eq. 1) and Accuracy (Eq. 2) denote the true negative rate and accuracy towards reporting respectively. Differences in both Specificity and Accuracy results between aligners or different parameter sets of the same aligner were assessed by building 95% bootstrap confidence intervals for the true difference. 10,000 bootstrap samples were used in building the interval estimates. Results were deemed significant at the 5% significance level if the associated 95% confidence interval (CI) for the differences failed to contain zero.

### Implementation and availability of CADBURE

We implemented the CADBURE algorithm as a Perl script. For rapid comparison, input files were required to be in Binary Alignment format (BAM) sorted by read name. The comparison involved collection of alignment information in the hash table, which provided a faster way of data search and retrieval. The comparison of tables was performed as per the scenarios 1–8 as described in Fig. 1. The CADBURE script is available at (http://cadbure.sourceforge.net/).

### Data and pre-processing

RNA-Seq was carried out as described^17^. Briefly, 6 libraries consisted of 3 biological replicates for each of two cell types (epithelial cells and fiber cells) from newborn FVB/N mouse lenses. Libraries were prepared according to the Illumina® TruSeq™ RNA Sample Preparation Kit v3 with mRNAs being poly (A) selected. Sequencing was performed using an Illumia HiSeq 2000 platform to produce 51 base long, single-ended reads. Read quality was assessed using Prinseq^29^, FastQC (http://www.bioinformatics.babraham.ac.uk/projects/fastqc/), and CLC Genomics Workbench. CLC Genomics Workbench was used to trim poor quality reads (Phred < 28), ambiguous poly (N) tails, poly (A/T) tails, adapters and primers.

### Spliced alignment to reference genome

The spliced alignment of final clean reads was performed against the mouse reference genome (ENSEMBL release 72^24^) with the guidance of the gene annotation model (GTF; ENSEMBL release 72). The known SNP data in variant call format (VCF; ENSEMBL release 72) was used in the case of SNP tolerant alignment. For GSNAP^9^ alignment (version 2013-09-30.v2), the known splice site information was extracted from GTF file using the GMAP utility and provided to the aligner with option (-s). For SNP tolerant alignment in GSNAP, SNP information was obtained from the VCF file using the GMAP utility and provided to the aligner with option (-v). Reads filtered by Illumina’s Chastity filter were recognized and filtered by the option (--filter-chastity both) in GSNAP. For Tophat2 alignment (version 2.0.9), the option (-G) was used to utilize the GTF annotation file to aid in splice alignment. For CLC Genomics Workbench (version 6.5.1), the genome was annotated with the GTF file to aid in spliced alignment to the genes. Three different runs were executed for each aligner (GSNAP, Tophat2, and CLC Genomics Workbench) in addition to the SNP tolerant alignment of GSNAP, by varying the mismatch levels (i.e., 0, 1, and 2 mismatches). Mapping results were obtained in Sequence Alignment Format (SAM) from all the aligners. Samtools^28^ was used to count the total mapped reads with the options (-c -F 4). Uniquely and non-uniquely mapped reads were identified by value to the SAM flag NH:i (Number of Hits; value 1 for unique mappings and value 2 or more for ambiguous mappings). All RNA-Seq bam files used in our data analysis are available on http://sourceforge.net/projects/cadbure/files/?source=navbar.

## Additional Information

**How to cite this article**: Kumar, P.K.R. *et al.* CADBURE: A generic tool to evaluate the performance of spliced aligners on RNA-Seq data. *Sci. Rep.* **5**, 13443; doi: 10.1038/srep13443 (2015).

## Supplementary Material

Supplementary Information

Supplementary Information

Supplementary Information
