# Supplementary material for: CADBURE: A generic tool to evaluate the performance of spliced aligners on RNA-Seq data
Source: Sci Rep. 2015 Aug 25;5:13443. doi: 10.1038/srep13443 (PMC4548254; doi:10.1038/srep13443)
Supplement: Supplementary Information [file srep13443-s1.pdf]

Supplemental Information

for

CADBURE: A generic tool to evaluate the performance of spliced aligners on RNA-Seq data.

Praveen Kumar Raj Kumar, Thanh V. Hoang, Michael L. Robinson, Panagiotis A. Tsonis, Chun Liang

Contents

1. Supplemental Results and Discussion 1
2. Supplemental Methods 3
3. Supplemental References 5
4. Supplemental Tables 7
5. Supplemental Figures 17

1. Supplemental Results and Discussion

Evaluation of methods used for RNA-Seq based differential expression analysis

The absolute value of gene expression counts were obtained from the optimal alignment result selected by CADBURE. Since CLC Genomics Workbench forbids the use of third party alignment results for RNA-Seq analysis, the CLC unique mapping results with maximum 2 mismatches provided the gene expression result for both the CLC versions of the Baggerley test (version 6.5.1) and EDGE (version 7.0), the latter being similar to that used in edgeR[1]. For DESeq and DESeq2, the count of reads mapped to genic regions were estimated using HTSeq[2] package. For Cuffdiff2, we followed the procedure described elsewhere[3].

As shown in Supplemental Figure S4, out of the five methods, CLC's Baggerley test predicted the most differentially expressed genes (DEGs) (i.e., 7,481 genes, with adjusted p value < 0.01 and fold change >= 2). This was followed by DESeq2 with 5,152 DEGs, CLC's EDGE with 4,991 DEGs, Cuffdiff2 with 4,165 DEGs and finally DESeq with only 1,856 DGEs. DESeq contained the most commonly shared DEGs (99.62%, shared by all 5 methods), whereas CLC's Baggerley contained the lowest number of shared DEGs (43.26%; Supplemental Fig. S4). For DEGs shared by two methods, DESeq2 and CLC's EDGE shared the most number of DEGs (i.e., 4,332 genes), followed by DESeq2 and Cuffdiff2 sharing 2,989 DEGs (for a full list see Supplemental Table S9). Even though DESeq predicted only 1,856 DEGs (adjusted p value < 0.01 and Fold change >= 2), 1,831 of these DGEs are shared with DESeq2 (Supplemental Fig. S4; Supplemental Table S9). When comparing sets of three methods, DESeq2, CLC's EDGE and Cuffdiff2 shared the most number of DEGs (i.e., 2,754 genes) and the least number of shared DEGs for three methods is among Cuffdiff2, CLC's Baggerley and DESeq (i.e., 413 genes; Supplemental Fig. S4 and Supplemental Table S9).

Performance assessment using receiver operating characteristic (ROC) curve for differentially expressed gene detection

The prediction performance and ability of each method were then evaluated in terms of the tradeoff between specificity and sensitivity. The calculation of specificity and sensitivity depends on the estimation of true-positive (TP), true-negative (TN), false-positive (FP) and false-negative (FN) genes among the predicted DEGs. The formula established by De Smet et al[4] and Storey and Tibshirani[5] permitted the identification of TP, FP, TN and FN among the DEGs as had originally been described for microarray experiments (details in methods). This formula involves

finding λ, which is described as the point after which the adjusted p-values becomes uniformly distributed. In short, this means the point after which none of the genes can be identified as a DEG significantly. In our study, we observed noises (peak at high p value) in the distribution for all tested methods except DESeq2. Hence, we removed high p-value peaks to find the p values uniformly distributed after 0.2, which forms our λ estimation. With this, we calculated the sensitivity (also called the true-positive rate) and specificity (1-specificity is called false-positive rate). Plotting the true-positive rate against false-positive rate at all possible rejection levels $\alpha = p_i$ (where i goes from 0 to 1) is called ROC (Receiver operating characteristic) curve[6]. A ROC curve reveals the tradeoff between true-positive rates and false-positive rates, which here is significantly highest for DESeq, followed by DESeq2, CLC's EDGE, Baggerely, and Cuffdiff2 (Supplemental Fig. S5 a). The area under the ROC (AUC) curve was computed with the trapezoid equation and was significantly higher for DESeq compared to the other methods (Supplemental Fig. S5 a). This suggests that there is better balance between true-positive rate and false-positive rate in DESeq versus the other methods with our data sets. To find the error rate for the AUC, we computed the error for five different fold changes 1.5, 1.75, 2, 2.25, and 2.5 (Supplemental Fig. S5 b). The result confirmed that DESeq performs better in comparison with other methods at all fold change levels. Surprisingly, the earlier version of DESeq exhibited a better balance between the true-positive rate and false-positive rate in comparison to the recently updated DESeq2, after noise (peaks at high p-value 1, 0.88-0.89) removal for DESeq. Even though CLC's Baggerley test revealed the highest number of potential false positives among five tools (Supplemental Fig. S4), the Baggerley test's AUC exceeded that of Cuffdiff2 (Supplemental Fig. S5 a). This may reflect the Baggerley test's better balance with more true positives.Cuffdiff2 performed the worst in comparison to other methods evaluated in terms of balance between the true-positive rate and false-positive rate (Supplemental Fig. S5 a, b). Surprisingly, DESeq initial version performed better than DESeq2 after noise removal (see **Methods**), and DESeq2's performance decreased with increasing the threshold of fold change past a value of 1.5 (Supplemental Fig. S5 b). This trend may be attributed to the behavior that DESeq2 assesses lower p values (< 0.01) to all the genes with greater fold change as we observed in this study.

Verification with RT-qPCR

In total, the expression level and differential expression profile of 18 genes were selected for verification of different DEG analysis methods with real time quantitative PCR (RT–qPCR; data provided by[7]). These genes were selected based on their biological significance in the mouse lens

development. For convenience, genes were grouped into three sections, according to their fold change levels, with positive and negative fold change indicating the gene up-regulation in fiber and epithelial cells respectively (Supplemental Fig.S6). Supplemental Figure S6a illustrated the odd behavior of CLC's Baggerely test, being the only test which did not agree with the qPCR results and other tests in the direction of fold change. This could result from the lack of normalization for sequencing depth across the samples. For instance, the DESeq size factor utility found that the biological replicates for epithelial cells were not homogenous in the sequencing depth. The size factors (as known as sequencing depth) obtained were 0.98, 2.15, 2.01, 0.68, 0.65 and 0.56 for E1, E2, E3, F1, F2 and F3, respectively, where E stands for epithelial cells and F stands for fiber cells. This non-homogeneity in the sequencing depth between samples needs to be normalized by the statistical tests for differential gene expression, and CLC's Baggerley test lacks this normalization. Among the five methods, DESeq2 and DESeq agree best (overlap of error rates) with qPCR expression in terms of fold change level, followed by CLC's EDGE and Cuffdiff2 (Supplemental Fig. S6 b, c).

2. Supplemental Methods

Identification of differentially expressed genes

To identify differentially expressed genes (DEGs) between lens epithelial cells and lens fiber cells, five different statistical methods: DESeq[2] and DESeq2[2], Cuffdiff2[8], and two versions of CLC Genomics Workbench - Baggerley test in version 6.5.1 and Empirical Analysis of Differential Gene Expression (EDGE) in version 7 were utilized. The raw counts of mapped reads to the genes were used for all the statistical methods except Cuffdiff2 which requires a normalized measure of gene count RPKM (Reads Per Kilo base per Million reads) that was obtained using Cufflinks 2.1[3]. The HTSeq package[2] was used to construct a gene count table/matrix used for DESeq and DESeq2. For CLC, which forbids third party count matrices, the matrix produced within CLC was used. For all the methods, significant DEGs were identified with false discovery rate (FDR) corrected p-value. A Venn diagram of the common genes was drawn using the software package VennDiagram[9] developed in R (*http://cran.r-project.org/*).

Evaluating the performance of statistical methods

Statistical methods for discriminating DEG analyses work on rejecting the null hypothesis, that genes are not truly differentially expressed. In the process, the methods delineate the conditions of the genes with p-value, probability of obtaining a test statistic when the null hypothesis is

true. We evaluated the ability of each method analyzed in terms of the tradeoff between specificity and sensitivity. Calculation of specificity and sensitivity depends on the estimation of true-positive (TP), true-negative (TN), false-positive (FP) and false-negative (FN) genes, which in turn requires the identification of genes that are not actually differentially expressed (i.e., for which the null hypothesis is true) and that are actually differentially expressed (i.e., for which the null hypothesis is false). This was accomplished using formulas established by De Smet et al[4] and Storey et al[5]. De Smet and co-worker's approach involves finding N_0, the number of genes that are not actually differentially expressed, and N_1, the number of genes that are actually differentially expressed. Their definition of N_0 is as follows,

$$N_0 = \lim_{\lambda \to 1} \frac{number\ of\ genes\ with\ p\ value > \lambda}{1-\lambda} \qquad \text{(Eq. 3)}$$

Here λ is the value after which the FDR adjusted p-values distribution becomes uniform. A uniform distribution of p-values is achieved for all the methods after removing the noise (peaks of high p-values). Once N_0 is estimated, N_1 can be derived by subtracting N_0 from N, the total number of expressed genes, i.e., $N_1 = N - N_0$. Next, let ϵ be the number of genes declared differentially expressed at a rejection p value α. Then

$$\text{TP} = \epsilon - \alpha N_0 \qquad \text{(Eq. 4)}$$

$$\text{FP} = \alpha N_0 \qquad \text{(Eq. 5)}$$

$$\text{TN} = (1-\alpha) N_0 \qquad \text{(Eq. 6)}$$

$$\text{FN} = N_1 - \epsilon + \alpha N_0 \qquad \text{(Eq. 7)}$$

$$\text{Sensitivity} = TP/(TP+FN) \qquad \text{(Eq. 8)}$$

Performance assessment using ROC curve

Specificity and Sensitivity of the methods were calculated using Equation 1 and 8, where Equations 1 and 2 are described in the main content of this paper. The ability of each method to identify actual DEGs was evaluated using the receiver operating characteristic (ROC) curve[10], a popular method to quantify method ability (For example see[11]). A ROC curve is obtained by plotting the true positive rate (sensitivity) versus the

false positive rate (1−specificity) at all possible rejection levels α=pi (i = 0,….1). The area under the ROC for each method was calculated using the trapezoid equation[12] by a PERL script. The scripts (PERL and R) developed for all the statistical analysis are available here (*http://cadbure.sourceforge.net/*).

Validation of RNA-seq results with RT-qPCR

The DEGs that disagree in fold change among five statistical packages (DESeq, DESeq2, Cuffdiff2, CLC's Baggerley test and EDGE) were selected based on their biological importance in the lens and verified with quantitative reverse transcription-polymerase chain reaction (RT-qPCR). The procedures for RT-qPCR are as described previously[7]. Briefly, cDNAs for the selected genes were synthesized using a reverse transcription kit (Invitrogen) according to the manufacturer's instructions. qPCR assay study was conducted; and the quantification cycle (Cq, also commonly called the Ct) was obtained and ΔCq value was calculated by $Cq_{(gene)} - Cq_{(GAPDH)}$. For fold-change expression, ΔΔCq was first calculated by subtracting the mean of ΔCq values of fiber samples from the mean ΔCq values of epithelial samples; and then converted to $2^{(-\Delta\Delta Cq)}$. The data were expressed as mean ± standard error of the mean (SEM).

3. Supplemental References

1. Robinson, M., McCarthy, D. & Smyth, G. EdgeR: a bioconductor package for differential expression analysis of digital gene expression data. *Bioinformatics* **26,** 139–140 (2010).
2. Anders, S. & Huber, W. Differential expression analysis for sequence count data. *Genome Biol* **11,** R106 (2010).
3. Trapnell, C. *et al.* Differential gene and transcript expression analysis of RNA-seq experiments with TopHat and Cufflinks. *Nat. Protoc.* **7,** 562–578 (2012).
4. De Smet, F. *et al.* Balancing false positives and false negatives for the detection of differential expression in malignancies. *Br. J. Cancer* **91,** 1160–1165 (2004).
5. Storey, J. D. & Tibshirani, R. Statistical significance for genomewide studies. *Proc. Natl. Acad. Sci.* **100,** 9440–9445 (2003).
6. Dawson, B. & Trapp, R. G. *Basic & Clinical Biostatistics*. (Appleton & Lange, 1994).

7. Hoang, T., Raj Kumar, P. K., Sutharzan, S., Tsonis, P. & Liang, C. Comparative transcriptome analysis of epithelial and fiber cells in newborn mouse lenses by RNA sequencing. *Mol. Vis.* **20**, 1491-1517 (2014).

8. Trapnell, C. *et al.* Differential analysis of gene regulation at transcript resolution with RNA-seq. *Nat. Biotechnol.* **31,** 46–53 (2013).

9. Chen, H. & Boutros, P. C. VennDiagram: a package for the generation of highly-customizable Venn and Euler diagrams in R. *BMC Bioinformatics* **12,** 35 (2011).

10. Hanley, J. A. & McNeil, B. J. The meaning and use of the area under a receiver operating characteristic (ROC) curve. *Radiology* **143,** 29–36 (1982).

11. Rapaport, F. *et al.* Comprehensive evaluation of differential gene expression analysis methods for RNA-seq data. *Genome Biol.* **14,** R95 (2013).

12. Fawcett, T. An introduction to ROC analysis. *Pattern Recognit. Lett.* **27,** 861–874 (2006).

13. Milne, I. *et al.* Using Tablet for visual exploration of second-generation sequencing data. *Brief. Bioinform.* **14,** 193–202 (2013).

4. Supplemental Tables

Supplemental Table S1. Counts of raw and trimmed reads.

Sample ID	Sequencing type	Raw Reads	Trimmed Reads	Avg. Length after Trim
Epithelial Replicate 1 (E1)	RNA-seq	33,174,286	30,524,674	47
Epithelial Replicate 2 (E2)	RNA-seq	29,919,226	27,392,629	46.9
Epithelial Replicate 3 (E3)	RNA-seq	29,965,660	28,203,944	46.9
Fiber Replicate 1 (F1)	RNA-seq	28,652,759	27,019,907	46.8
Fiber Replicate 2 (F2)	RNA-seq	30,661,663	27,760,247	46.9
Fiber Replicate 3 (F3)	RNA-seq	24,833,352	22,735,990	46.8

Reads are trimmed based on the low quality, adapters, primers, poly (A)/ (T) and ambiguous poly (N) tails. Read length before trimming was 51 nt uniformly.

Supplemental Table S2. Percentage of trimmed reads mapped to reference by parameter set.

Protocols	E1	E2	E3	F1	F2	F3
GSNAP-SNP-tol 2 mismatch	96.36%	99.61%	96.79%	95.54%	99.04%	99.54%
GSNAP 2 mismatch	95.53%	99.49%	96.60%	95.47%	98.83%	99.41%
TopHat2 2mM	79.90%	95.72%	92.54%	92.25%	92.73%	95.51%
GSNAP-SNP-tol 1 mismatch	77.58%	94.06%	90.94%	90.83%	91.21%	94.02%
TopHat2 1 mismatch	75.64%	93.56%	90.40%	90.28%	90.25%	93.35%
GSNAP 1 mismatch	74.87%	93.12%	89.95%	90.08%	89.98%	93.12%
CLC 2 mismatch	69.04%	90.17%	86.92%	87.76%	87.11%	90.70%
CLC 1 mismatch	65.55%	88.21%	84.98%	85.85%	84.79%	88.63%
GSNAP-SNP-tol 0 mismatch	66.51%	82.94%	80.05%	80.27%	80.27%	83.05%
GSNAP 0 mismatch	60.55%	80.00%	77.11%	77.27%	76.50%	79.71%
TopHat2 0 mismatch	59.15%	79.47%	76.36%	76.79%	75.74%	79.07%
CLC 0 mismatch	53.07%	75.38%	72.41%	73.18%	71.63%	75.34%

Table is sorted by the values in E1. GSNAP-SNP-tol: GSNAP with SNP tolerance.

Supplemental Table S3. Absolute counts for Scenarios 1 to 8 in the pairwise comparison of alignment results between GSNAP with SNP tolerance and TopHat2 identified by CADBURE.

Scenario	E1		E2		E3		F1		F2		F3	
	GSNAP with SNP tolerance	TopHat2	GSNAP with SNP tolerance	TopHat2	GSNAP with SNP tolerance	TopHat2	GSNAP with SNP tolerance	TopHat2	GSNAP with SNP tolerance	TopHat2	GSNAP with SNP tolerance	TopHat2
1.	12,656,645	12,656,645	20,453,448	20,453,448	19,909,201	19,909,201	19,696,509	19,696,509	19,167,240	19,167,240	16,987,204	16,987,204
2.	1,016	46,266	2,503	113,815	2,372	102,035	1,816	89,125	1,733	85,114	1,657	76,316
3.	476,054	430,804	454,463	343,151	426,590	326,927	469,901	382,592	439,388	356,007	355,064	280,405
4.	244,611	244,611	84,196	84,196	105,689	105,689	54,135	54,135	86,655	86,655	51,928	51,928
5.	149,834	5,847,677	93,329	2,900,719	90,586	3,221,787	468,732	1,470,479	383,647	2,349,866	296,568	1,446,903
6.	4,577,108	2,916	911,936	1,594	1,054,947	1,884	784,515	1,401	1,578,877	1,645	814,537	1,272
7.	5,010,825	5,010,825	2,228,921	2,228,921	2,340,707	2,340,707	2,762,823	2,762,823	3,311,726	3,311,726	2,573,666	2,573,666
8.	450,197	825	156,408	355	147,824	465	105,848	221	175,913	303	103,243	223
True Positives	17,709,807	13,090,365	21,819,847	20,798,193	21,390,738	20,238,012	20,950,925	20,080,502	21,185,505	19,524,892	18,156,805	17,268,881
False Positives	395,461	6,138,554	180,028	3,098,730	198,647	3,429,511	524,683	1,613,739	472,035	2,521,635	350,153	1,575,147
True Negatives	11,308,699	5,161,484	5,286,048	2,322,605	5,710,318	2,431,758	4,339,150	3,231,776	5,837,505	3,695,676	4,123,812	2,870,457
Specificity	0.9662	0.4568	0.9671	0.4284	0.9664	0.4149	0.8921	0.667	0.9252	0.5944	0.9217	0.6457
Accuracy	0.9866	0.7483	0.9934	0.8818	0.9927	0.8686	0.9797	0.9353	0.9828	0.902	0.9845	0.9275

Supplemental Table S4. Specificity and Accuracy differences in the pairwise comparison of alignment results between GSNAP with SNP tolerance and TopHat2 identified by CADBURE.

Sample	Specificity difference between GSNAP with SNP tolerance and TopHat2 using Bootstrap		Accuracy difference between GSNAP with SNP tolerance and TopHat2 using Bootstrap	
	Mean(SE)	95% CI	Mean (SE)	95% CI
E1	0.5094(0.0002)	(0.0129,0.4967)	0.2382(0.0001)	(0.0060,0.2323)
E2	0.5386(0.0002)	(0.0137,0.5252)	0.1116(0.0001)	(0.0028,0.1088)
E3	0.5515(0.0002)	(0.0140,0.5377)	0.1241(0.0001)	(0.0032,0.1210)
F1	0.2252(0.0003)	(0.0059,0.2196)	0.0444(0.0001)	(0.0012,0.0433)
F2	0.3308(0.0002)	(0.0085,0.3225)	0.0808(0.0001)	(0.0021,0.0788)
F3	0.2761(0.0003)	(0.0071,0.2692)	0.0571(0.0001)	(0.0015,0.0556)

Supplemental Table S5. Absolute counts for Scenarios 1 to 8 in the pairwise comparison of alignment results between GSNAP without SNP tolerance and TopHat2 identified by CADBURE.

Scenario	E1		E2		E3		F1		F2		F3	
	GSNAP	TopHat2	GSNAP	TopHat2	GSNAP	TopHat2	GSNAP	TopHat2	GSNAP	TopHat2	GSNAP	TopHat2
1.	14,971,258	14,971,258	21,008,010	21,008,010	20,565,863	20,565,863	20,017,045	20,017,045	19,818,170	19,818,170	17,310,903	17,310,903
2.	1,117	47,579	2,826	116,173	2,649	104,546	1,940	90,187	1,849	85,968	1,747	77,275
3.	629,095	582,633	580,272	466,925	555,077	453,180	537,226	448,979	574,692	490,573	449,621	374,093
4.	775,756	775,756	134,328	134,328	217,101	217,101	77,843	77,843	233,189	233,189	105,862	105,862
5.	69,808	2,844,938	69,717	2,168,078	65,294	2,323,144	242,396	1,057,464	170,207	1,415,119	89,216	973,327
6.	4,317,766	6,755	879,574	3,409	997,607	3,689	739,439	2,723	1,494,374	3,508	771,349	2,568
7.	5,090,220	5,090,220	2,252,248	2,252,248	2,365,688	2,365,688	2,988,983	2,988,983	3,524,903	3,524,903	2,780,830	2,780,830
8.	460,912	1,456	156,559	640	151,858	776	132,324	397	201,304	566	118,659	411
True Positives	19,918,119	15,560,646	22,467,856	21,478,344	22,118,547	21,022,732	21,293,710	20,468,747	21,887,236	20,312,251	18,531,873	17,687,564
False Positives	846,681	3,668,273	206,871	2,418,579	285,044	2,644,791	322,179	1,225,494	405,245	1,734,276	196,825	1,156,464
True Negatives	8,396,070	5,161,484	4,576,885	2,322,605	4,840,690	2,431,758	4,178,771	3,231,776	5,141,326	3,695,676	3,872,816	2,870,457
Specificity	0.9084	0.5846	0.9568	0.4899	0.9444	0.479	0.9284	0.7251	0.9269	0.6806	0.9516	0.7128
Accuracy	0.971	0.8496	0.9924	0.9078	0.9895	0.8987	0.9875	0.9508	0.9852	0.9326	0.9913	0.9467

Supplemental Table S6. Specificity and Accuracy differences in the pairwise comparison of alignment results between GSNAP without SNP tolerance and TopHat2 identified by CADBURE.

Sample	Specificity difference between GSNAP and TopHat2 using Bootstrap		Accuracy difference between GSNAP and TopHat2 using Bootstrap	
	Mean(SE)	95% CI	Mean (SE)	95% CI
E1	0.3238(0.0002)	(0.0083,0.3158)	0.1214(0.0001)	(0.0031,0.1183)
E2	0.4669(0.0002)	(0.0119,0.4552)	0.0847(0.0001)	(0.0022,0.0825)
E3	0.4654(0.0002)	(0.0119,0.4537)	0.0909(0.0001)	(0.0023,0.0886)
F1	0.2034(0.0002)	(0.0053,0.1983)	0.0367(0.0001)	(0.0010,0.0358)
F2	0.2463(0.0002)	(0.0064,0.2402)	0.0526(0.0001)	(0.0014,0.0513)
F3	0.2388(0.0003)	(0.0062,0.2329)	0.0445(0.0001)	(0.0012,0.0434)

Supplemental Table S7. Absolute counts for Scenarios 1 to 8 in the pairwise comparison of alignment results between GSNAP without SNP tolerance and GSNAP with SNP tolerance identified by CADBURE.

Scenario	E1		E2		E3		F1		F2		F3	
	GSNAP	GSNAP with SNP tolerance	GSNAP	GSNAP with SNP tolerance	GSNAP	GSNAP with SNP tolerance	GSNAP	GSNAP with SNP tolerance	GSNAP	GSNAP with SNP tolerance	GSNAP	GSNAP with SNP tolerance
1.	15,429,348	15,429,348	21,526,125	21,526,125	20,995,509	20,995,509	20,926,919	20,926,919	20,644,600	20,644,600	17,932,867	17,932,867
2.	170	26,334	380	1,923	343	2,694	162	759	136	6,028	114	1,752
3.	1,355,343	1,329,179	217,952	216,409	264,522	262,171	146,698	146,101	410,804	404,912	173,825	172,187
4.	683,815	683,815	111,937	111,937	154,450	154,450	72,254	72,254	198,949	198,949	91,209	91,209
5.	3,283,606	397,422	817,561	111,685	987,450	122,676	469,307	310,428	1,033,825	343,381	529,549	280,907
6.	12,518	239,170	772	31,796	1,317	51,885	549	19,147	4,167	59,670	1,134	28,036
7.	7,998,647	7,998,647	4,465,189	4,465,189	4,718,005	4,718,005	3,868,343	3,868,343	4,797,944	4,797,944	3,591,908	3,591,908
8.	1	26,446	11	3,298	9	4,863	0	1,500	1	5,736	1	2,355
True Positives	16,797,209	16,997,697	21,744,849	21,774,330	21,261,348	21,309,565	21,074,166	21,092,167	21,059,571	21,109,182	18,107,826	18,133,090
False Positives	3,967,591	1,107,571	929,878	225,545	1,142,243	279,820	541,723	383,441	1,232,910	548,358	620,872	373,868
True Negatives	8,396,070	11,308,699	4,576,885	5,286,048	4,840,690	5,710,318	4,178,771	4,339,150	5,141,326	5,837,505	3,872,816	4,123,812
Specificity	0.6791	0.9108	0.8311	0.9591	0.8091	0.9533	0.8852	0.9188	0.8066	0.9141	0.8618	0.9169
Accuracy	0.8639	0.9623	0.9659	0.9917	0.9581	0.9898	0.979	0.9851	0.9551	0.9801	0.9725	0.9835

Supplemental Table S8. Specificity and Accuracy differences in the pairwise comparison of alignment results between GSNAP without SNP tolerance and GSNAP with SNP tolerance identified by CADBURE.

Sample	Specificity difference between GSNAP with SNP tolerance and GSNAP without SNP tolerance using Bootstrap		Accuracy difference between GSNAP with SNP tolerance and GSNAP without SNP tolerance using Bootstrap	
	Mean(SE)	95% CI	Mean (SE)	95% CI
E1	0.2317(0.0002)	(0.0059,0.2259)	0.0984(0.0001)	(0.0025,0.0959)
E2	0.1279(0.0002)	(0.0034,0.1247)	0.0259(0.0000)	(0.0007,0.0252)
E3	0.1442(0.0002)	(0.0038,0.1406)	0.0317(0.0000)	(0.0008,0.0309)
F1	0.0336(0.0002)	(0.0010,0.0327)	0.0061(0.0000)	(0.0002,0.0060)
F2	0.1076(0.0002)	(0.0029,0.1049)	0.0250(0.0000)	(0.0007,0.0244)
F3	0.0550(0.0002)	(0.0016,0.0537)	0.0110(0.0000)	(0.0003,0.0107)

Supplemental Table S9. Number of differentially expressed genes (DEGs) shared between methods.

Methods for detecting DEG	Predicted Numbers of DEG
DESeq2 and CLC EDGE	4332
DESeq2 and Cuffdiff2	2989
CLC EDGE and CLC Baggerley	2959
DESeq2 and CLC Baggerley	2945
CLC EDGE and Cuffdiff2	2837
DESeq2, CLC EDGE and Cuffdiff2	2754
DESeq2, CLC EDGE and CLC Baggerley	2704
DESeq2 and DESeq	1831
CLC Baggerley and Cuffdiff2	1641
DESeq and Cuffdiff2	1633
DESeq and CLC EDGE	1627
DESeq2, DESeq and Cuffdiff2	1620
DESeq2, DESeq and CLC EDGE	1619
DESeq2, CLC Baggerley and Cuffdiff2	1598
CLC EDGE, CLC Baggerley and Cuffdiff2	1583
DESeq2, CLC EDGE, CLC Baggerley and Cuffdiff2	1576
DESeq, CLC EDGE and Cuffdiff2	1467
DESeq2, DESeq, CLC EDGE and Cuffdiff2	1464
DESeq and CLC Baggerley	505
DESeq2, DESeq and CLC Baggerley	505
DESeq, CLC EDGE and CLC Baggerley	504
DESeq2, DESeq, CLC EDGE and CLC Baggerley	504
DESeq, CLC Baggerley and Cuffdiff2	413
DESeq2, DESeq, CLC Baggerley and Cuffdiff2	413
DESeq, CLC EDGE, CLC Baggerley and Cuffdiff2	412
DESeq2, DESeq, CLC EDGE, CLC Baggerley and Cuffdiff2	412

Table is sorted by the numbers of shared genes.

Supplemental Table S10. The difference of a DEG determined using TopHat2 alignment versus SNP-tolerant GSNAP alignment.

	id	baseMean	baseMean_E	baseMean_F	foldChange	log2FoldChange	Pval	padj
TOPHAT	Rpl12	3874.69299	3231.135684	4518.25	1.398347	0.483723	0.002078	0.008811
GSNAP with SNP tolerance	Rpl12	273.232588	337.0806039	209.3846	0.621171	-0.68694	0.009925	0.037304

Supplemental Table S11. An excel file containing the reads reported as uniquely mapped to gene Rpl12 by TopHat2 result.

Supplemental Table S12. An excel file containing the same reads as in S11 with the mapping records as reported in GSNAP with SNP tolerance.

5. Supplemental Figures

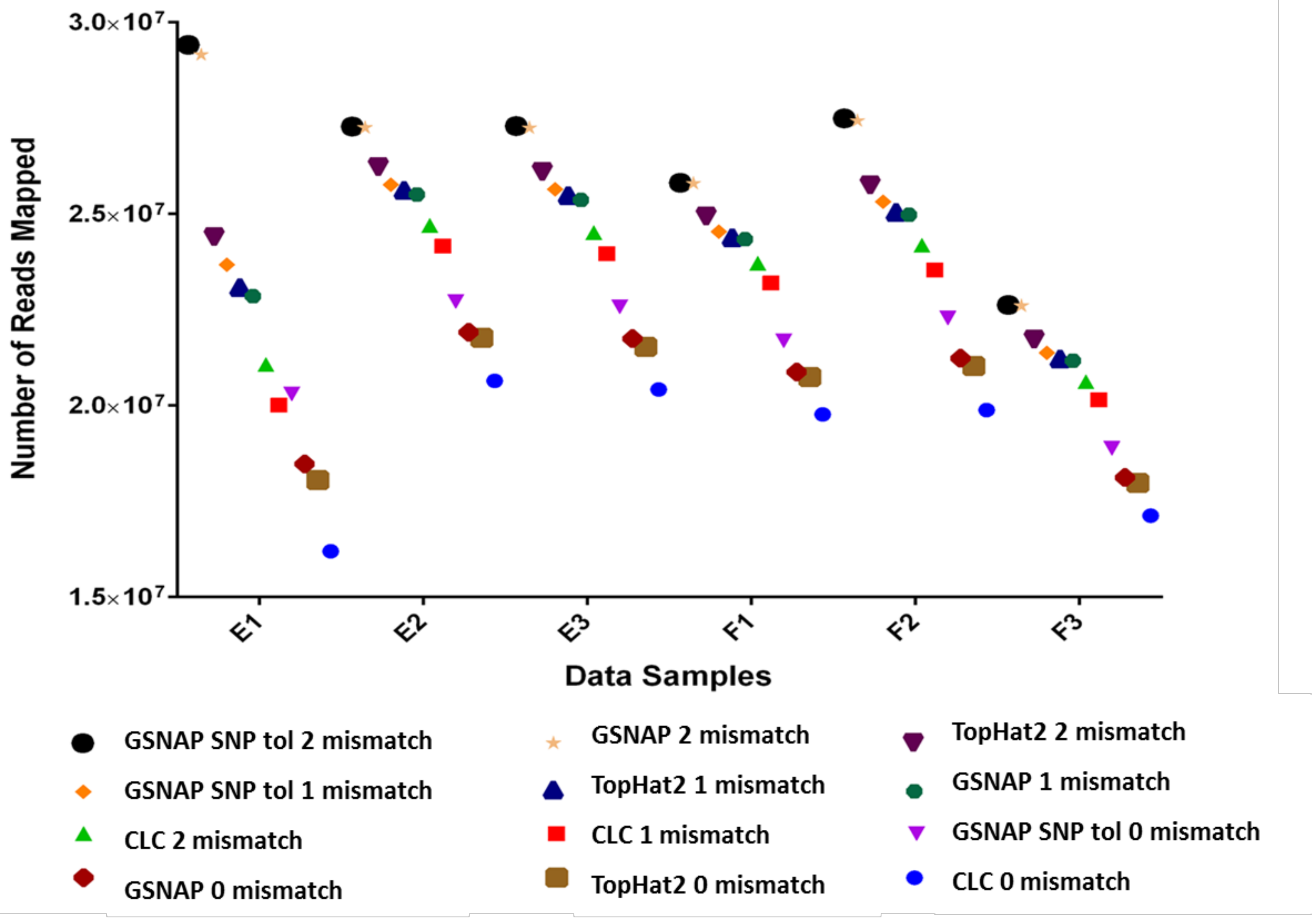

Supplemental Figure S1. Total mapped reads of all the 6 data samples by using twelve different mapping parameter-sets with three different aligners: GSNAP, TopHat2 & CLC Genomics Workbench.

The parameters varied are mismatch levels (i.e., 0, 1, 2 mismatches). E1, E2, E3 and F1, F2 and F3 are the three biological replicates of epithelial and fiber cells. The suffix mM next to a number indicates the allowed mismatch level in the mapping. The letters SNP-tol denotes SNP-tolerant alignment.

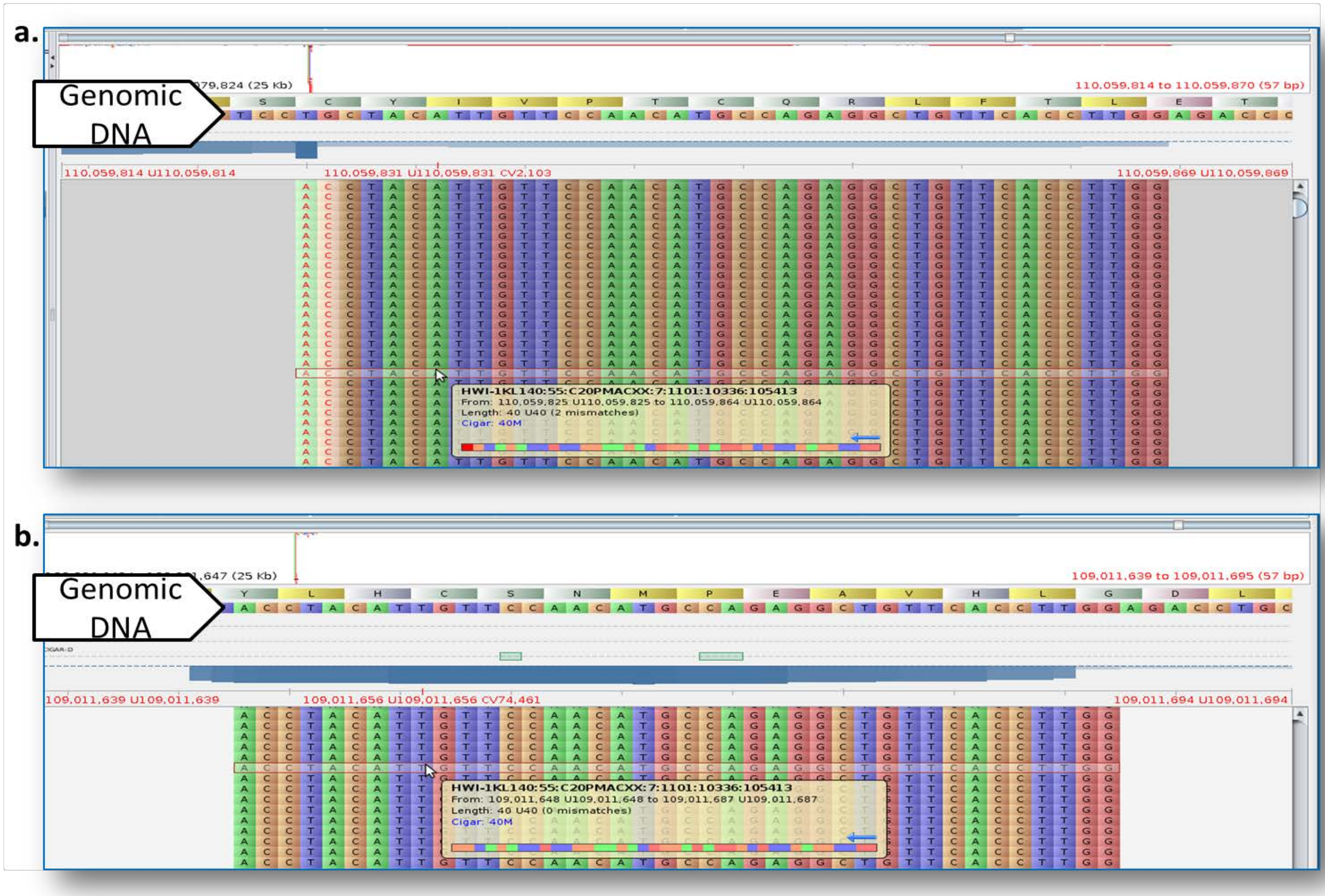

Supplemental Figure S2: An example of Scenario 4.

Read mapping (highlighted with red line) is identified as Scenario 4 by CADBURE and visualized in Tablet[13]. Tablet shows reads mapped against the mouse reference genome. The same read (name shown in popup) was reported as mapped uniquely to different genome locations by both GSNAP and TopHat2 aligners. (a). GSNAP mapped the highlighted 40 base read perfectly with no mismatches to Chromosome 11 from 109,011,648 to 109,011,687, whereas (b) TopHat2 mapped the same read with allowed two mismatches to Chromosome 7 from 110,059,825 to 110,059,864.

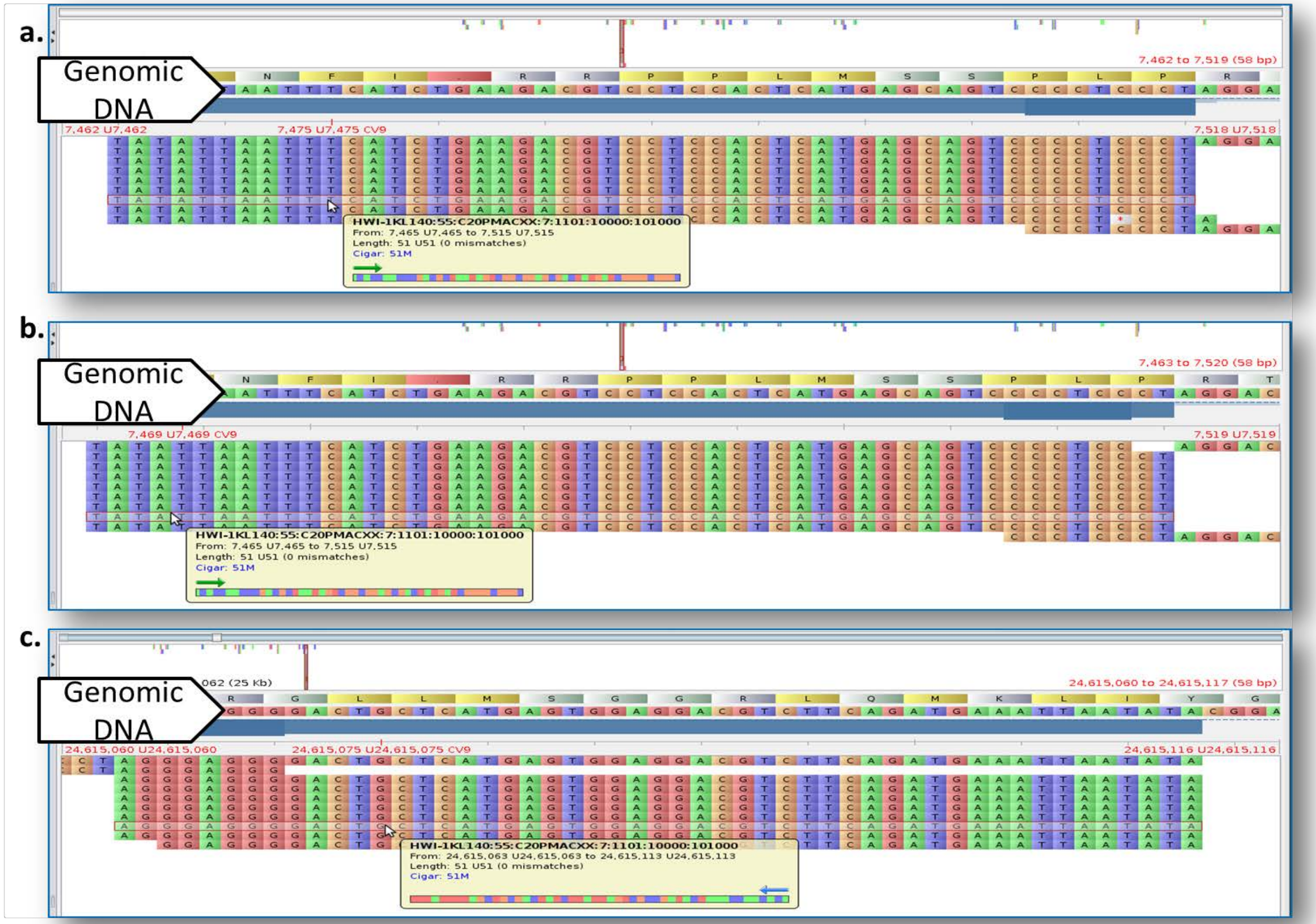

Supplemental Figure S3: An example of the Scenario 5.

Read mapping (highlighted with red line) is identified as Scenario 5 by CADBURE and visualized in Tablet[13]. Tablet shows reads mapped against the mouse reference genome. The same read (name shown in popup) was reported as mapped uniquely by TopHat2 and reported as mapped non-uniquely by GSNAP. (a). TopHat2 mapped the highlighted 51 base read with no mismatches to mitochondria from 7,465 to 7,515 and reported as unique mapping, whereas (b) GSNAP, in addition to mapping to mitochondria, also mapped the same read with reverse orientation and with no mismatches to Chromosome 1 from 24,615,063 to 24,615,663 (c).

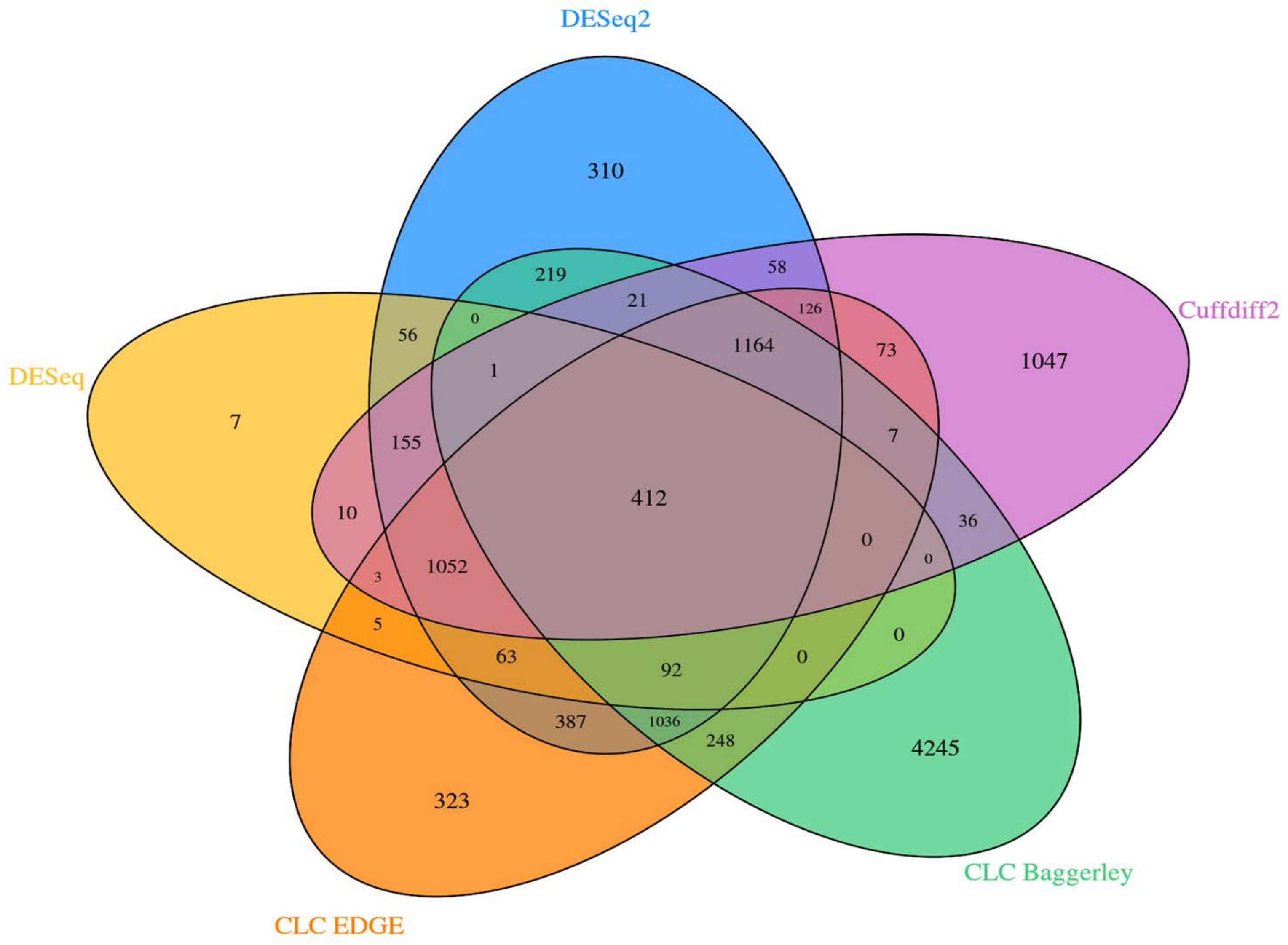

Supplemental Figure S4: A Venn diagram of the comparison of Differentially Expressed Genes (DEGs) identified by five different statistical methods: Cuffdiff2, DESeq, DESeq2, CLC Baggerely test and CLC EDGE.

The numbers represent the number of significant DEGs ($p < 0.01$ and fold change >= 2).

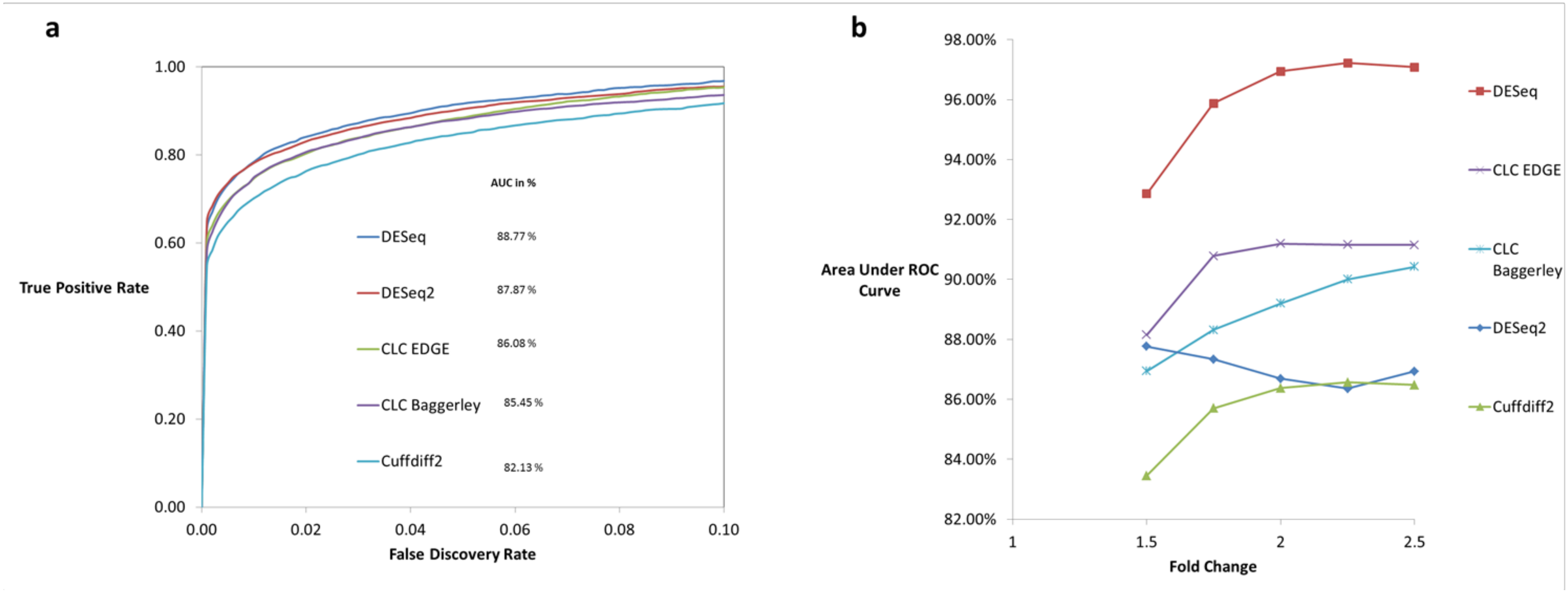

Supplemental Figure S5: Receiver operating characteristic (ROC) curve for the differentially expressed genes identified by five different statistical methods: Cuffdiff2, DESeq, DESeq2, CLC Baggerley test and CLC EDGE.

(a) ROC curve with all fold changes included. Fold-change was calculated based on the gene expression in the fiber cells relative to the epithelial cells. Negative values indicate expression lower in the fiber cells. Area under the curve (AUC) has shown for all the methods. (b) AUC for the five methods at five different fold changes 1.5, 1.75, 2, 2.25 and 2.5 of the differentially expressed genes.

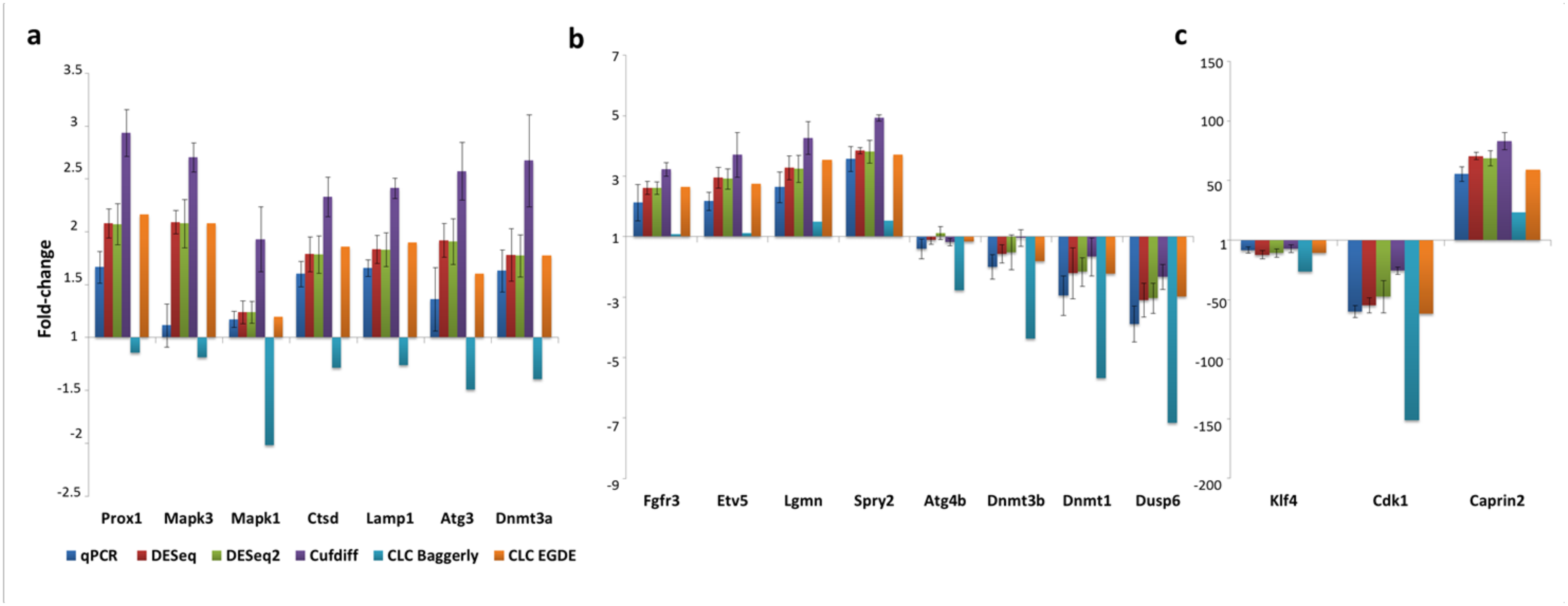

Supplemental Figure S6: Quantitative measurement of differentially expressed genes (DEGs) was determined by qRT–PCR for total 18 genes with a wide range of the expression levels as compared with DEGs identified by five different statistical methods: Cuffdiff2, DESeq, DESeq2, CLC Baggerely test and CLC EDGE.

The comparison for 18 genes is split into 3 groups a, b and c according to the range of fold changes of DEGs. Fold-change was calculated based on the gene expression in the fiber cells relative to the epithelial cells. Negative values indicate expression lower in the fiber cells. (**a**) Seven genes with fold changes less than +/- 4. (**b**) Eight with fold changes less than +/- 8. (**c**) Three genes with fold changes from +/- 6 to +/- 153.

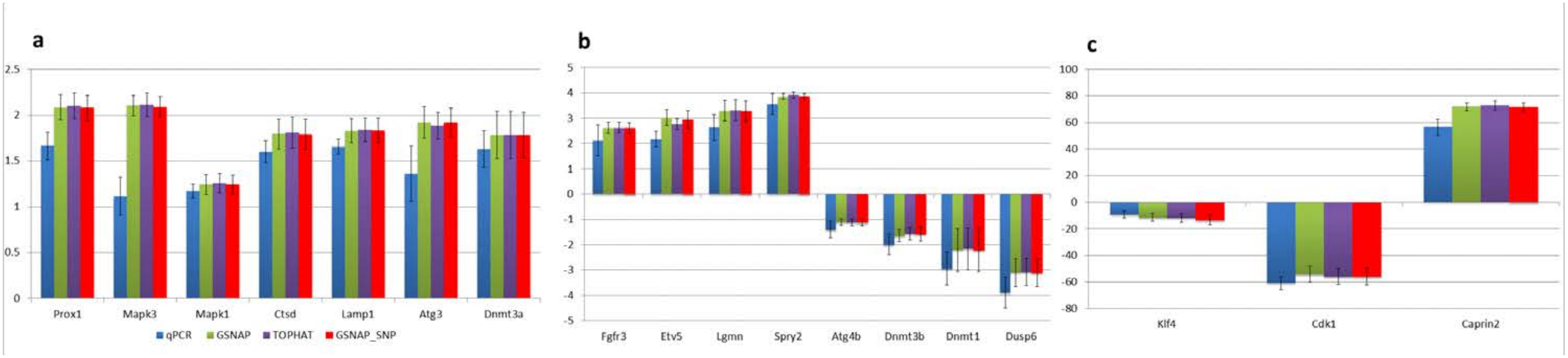

Supplemental Figure S7: The fold change of 18 differentially expressed genes (DEGs) as identified by DESeq for CADBURE selected alignment result (GSNAP with SNP tolerance), GSNAP and TopHat2 was compared with qRT–PCR.

The comparison for 18 genes is split into 3 groups a, b and c according to the range of fold changes of DEGs. Fold-change was calculated based on the gene expression in the fiber cells relative to the epithelial cells. Negative values indicate expression lower in the fiber cells. (a) Seven genes with fold changes less than +/- 4. (b) Eight with fold changes less than +/- 8. (c) Three genes with fold changes from +/- 6 to +/- 153.

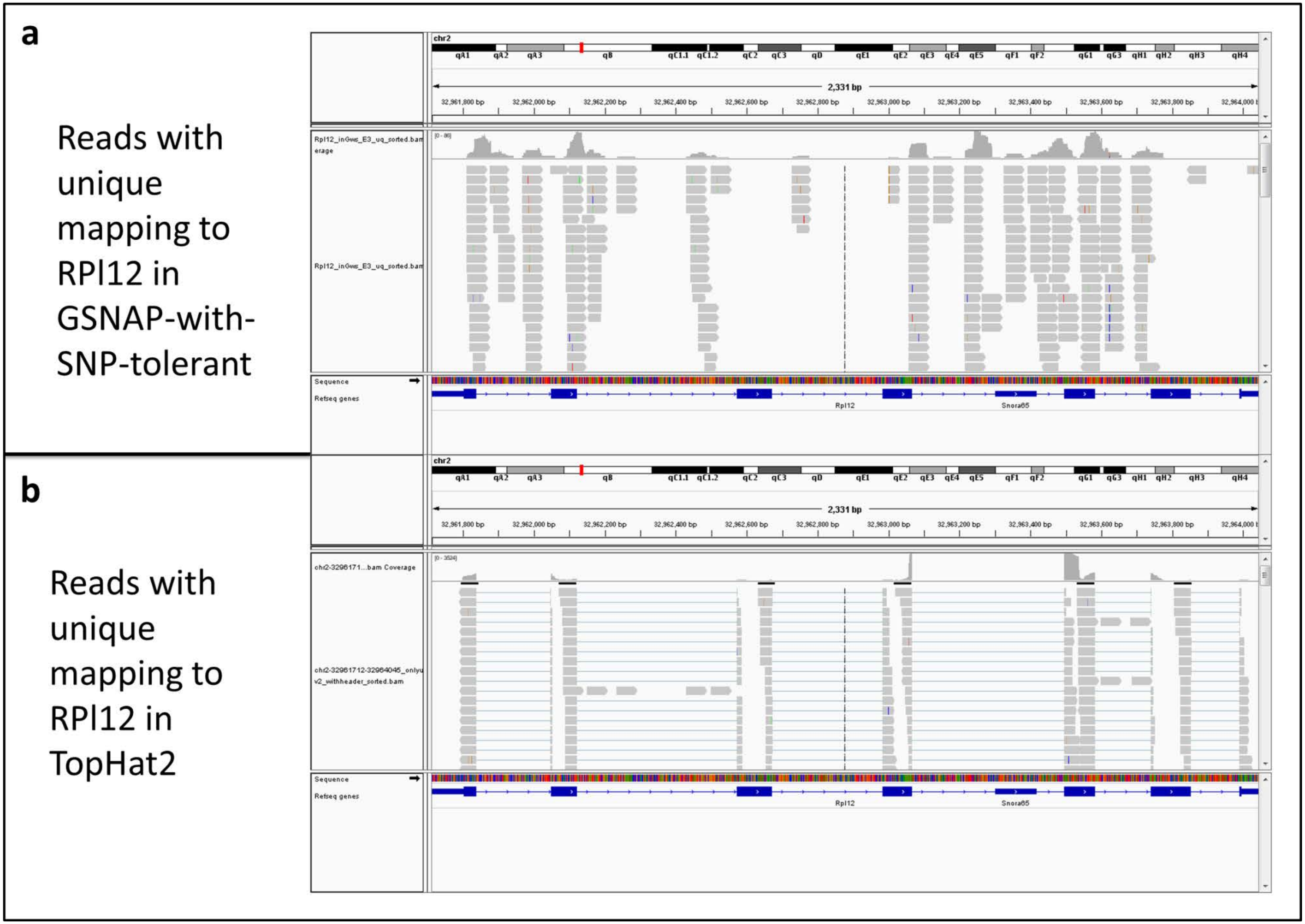

Supplemental Figure S8: IGV (Integrative Genome Viewer) snapshots of reads reported as uniquely aligned to gene RPI12 by GSNP with SNP tolerance (a: CADBURE selected alignment result) versus TopHat2 (b: Non-CADBURE result).
